# Oral Rehabilitation and Mental Health: Effects of the Renew Procedure on Depression Scores

**DOI:** 10.7759/cureus.85843

**Published:** 2025-06-12

**Authors:** Austin F Cross, William L Balanoff, Bernard Schayes, William Jayne

**Affiliations:** 1 Research and Development, Renew LLC, Centennial, USA; 2 Medical Affairs, Emblem Health, New York, USA; 3 Medicine, Northwell Health, New York, USA

**Keywords:** depression, implant-retained dentures, inflammation, mental health, oral-systemic connection, patient health questionnaire-9 (phq-9), periodontitis, social determinants of health (sdoh), systemic health

## Abstract

The Renew Procedure is an elective comprehensive surgical and prosthetic intervention designed to address severe oral health issues, particularly periodontitis, and by systematically eliminating sources of chronic inflammation in the mouth, overall systemic health may improve. Additionally, restoring functionality and aesthetics to the mouth can significantly enhance a patient’s quality of life. This study investigates whether improvements in oral health via the Renew Procedure correlate with measurable reductions in depressive symptoms, as assessed using the Patient Health Questionnaire-9 (PHQ-9), a pre- and post-operative assessment administered to patients undergoing full-mouth rehabilitation with the Renew Procedure. The results indicate a statistically significant decrease in PHQ-9 scores, suggesting that the intervention may contribute to improved mental health outcomes. These findings support the need for integrated healthcare approaches that recognize the interconnections between oral health, systemic inflammation, and mental well-being.

## Introduction

The Renew Procedure [1] is an advanced full-mouth rehabilitation technique that removes all periodontally compromised teeth, eliminates chronic infection, and places implant-retained prosthetic dentures. This intervention effectively resolves chronic oral inflammation while restoring functionality and aesthetics. Periodontitis, one of the most prevalent inflammatory diseases in humans, combined with tooth decay, is the most significant combined threat to dental health worldwide, and inflammation plays a significant role in exacerbating these two issues [2-4]. More than 47% of adults over 30 and approximately 70% of adults over 65 are affected by periodontitis; therefore, it is essential to look at the mechanisms causing this disease [5]. Chronic periodontitis is characterized by progressive destruction of the periodontal ligament and alveolar bone, and the disease process is driven by persistent inflammation triggered by bacterial biofilms, which can extend beyond the oral cavity and contribute to systemic conditions. This can lead to prominent bleeding, gum recession, and total loss of some/all teeth [6].

As periodontal disease develops further, additional concerns and symptoms, such as elevated blood pressure, can arise. Specifically, systolic blood pressure (SBP) can climb to dangerous, life-threatening levels, greater than or equal to 140 mmHg [7,8]. Further, untreated periodontal disease is associated with increased risks for diseases, including atherosclerosis [9], Parkinson’s disease [10], diabetes [1,11], coronary artery disease [12], male impotence, and more. Recent research also suggests a potential link to liver disease via gram-negative bacteria prevalent in both periodontitis and liver disease [13,14]. Given the established links between chronic periodontitis, inflammation, and depression, we hypothesized that patients undergoing the Renew Procedure would experience a significant reduction in depressive symptoms following treatment. This study assesses the changes in the Patient Health Questionnaire-9 (PHQ-9) scores pre- and post-surgery to determine whether full-mouth rehabilitation has a measurable impact on mental health.

Background

Periodontitis and the Renew Procedure

Recent studies have linked periodontitis to an increased risk of cardiovascular disease, diabetes, chronic kidney disease, and neurodegenerative disorders [9,10,12]. Inflammatory biomarkers such as C-reactive protein (CRP), interleukin-6 (IL-6), and tumor necrosis factor-alpha (TNF-α) are elevated in both periodontitis and depression, raising the possibility of a shared inflammatory pathway [15,16]. Recent studies indicate that individuals with generalized aggressive periodontitis have significantly higher PHQ-9 scores compared to those with chronic periodontitis or periodontally healthy controls [17]. However, the literature remains inconsistent, with some studies reporting no significant association between PHQ-9 scores and periodontal status [18]. This variability suggests that additional factors may mediate the periodontal and mental health relationship. These findings suggest that periodontal disease affects a person negatively beyond just oral health by also impacting a person’s overall systemic and holistic health.

Additionally, periodontal disease manifests in either a chronic or aggressive form (chronic periodontitis (CP) or aggressive periodontitis (AgP)), with the aggressive form diagnosed via rapid progression, early age onset, and specific patterns of tissue loss despite the absence of systemic disease [19]. Although CP and AgP exhibit similar clinical measures of alveolar bone reabsorption, probing depth, and attachment loss, studies indicate that AgP is associated with higher levels of pro-inflammatory mediators, such as TNF-α, and lower levels of cytokines like interleukin-2 (IL-2), which may play a role in immune regulation [20,21].

The standard treatment for both types of periodontitis begins with full-mouth debridement, which removes bacterial plaque and calculus to control infection and inflammation [22]. This is typically performed through scaling and root planing (SRP), a non-surgical intervention in which a dentist or dental hygienist removes subgingival calculus and plaque deposits. In some cases, systemic or locally administered antibiotics enhance the therapeutic outcomes [23].

Surgical intervention is necessary for advanced periodontitis cases resulting in total tooth loss. The Renew Procedure is a full-mouth rehabilitation approach that extracts all remaining teeth, removes periodontally compromised bone, eliminates oral and maxillofacial infections, and places anchored dental implants into the jawbone [1]. Implant-supported and implant-retained dentures are fitted onto these anchored implants, also known as abutments, providing the patient with structural support and improved oral function. After healing and minor adjustments to the dimensions and placements in the mouth, the result is usually a set of overdentures that snap on and off, requiring zero additional adhesives or bonding agents. Some patients occasionally only require a procedure for the lower denture, depending on the remaining quality and quantity of bone in both the maxilla and mandible.

PHQ-9

Recent research highlights a significant association between chronic oral conditions like periodontitis and elevated rates of depression, as measured by PHQ-9 [24]. PHQ-9 is a widely used diagnostic tool specifically designed to assess depression severity, offering a reliable framework for identifying depressive symptoms in clinical and research settings. The survey consists of nine questions, with responses ranging from 0 ("not at all") to 3 ("nearly every day"), based on the patient’s experiences over the preceding two weeks. A cumulative score is calculated to categorize the severity of depression: minimal or none (0-4), mild (5-9), moderate (10-14), moderately severe (15-19), and severe (20-27) [25]. Its ease of use and ability to capture longitudinal changes in depressive symptoms make it a valuable resource for assessing mental health across diverse patient populations, including those with chronic systemic conditions [25]. Moreover, PHQ-9 has been validated as an effective measure of depression in studies addressing the interplay between physical and mental health, with sensitivity to inflammatory and psychosocial factors​​ [26].

A study analyzing data from the National Health and Nutrition Examination Survey 2009-2014 found that individuals with severe depressive symptoms had higher odds of mild periodontitis compared to non-symptomatic individuals [27]. Additionally, those with mild to moderate depressive symptoms exhibited an increased number of missing teeth [27]. Another investigation revealed that each additional tooth lost was associated with an increase in depressive symptoms, as indicated by higher PHQ-9 scores and a greater likelihood of clinical depression (PHQ-9 score ≥10) [28]. A study focusing on the combined effects of smoking, drinking, and depression on periodontal disease reported a significant interaction between smoking and depression in relation to periodontitis risk. Specifically, among individuals experiencing depression, smoking was found to significantly increase the prevalence of periodontitis compared to non-smokers [29]. It is important that integrated healthcare approaches that simultaneously address both oral and mental health are emphasized moving forward.

A more recent study found no significant correlation between depression (measured via the PHQ-9 scale), periodontitis, and multiple periodontal conditions, including pocket depth, bleeding on probing, and clinical attachment loss [18]. This divergence in results raises critical questions about the utility of PHQ-9 in assessing mental health in populations with periodontal disease. This variability highlights the necessity of considering additional mediating factors, such as systemic inflammation, behavioral patterns, and social determinants of health (SDoH), which may confound observed relationships. Future research addressing these inconsistencies should involve larger, more diverse populations and integrate nuanced health and behavioral data.

SDoH

Understanding the relationship between periodontal disease and mental health requires moving beyond biological markers and individual behaviors towards considering broader contextual influences. Modern public health research increasingly acknowledges the role of SDoH in shaping health outcomes and disparities. These determinants encompass socioeconomic status, education, employment, access to healthcare, and community infrastructure. Furthermore, a wide array of data supports the relationship between SDoH and higher PHQ-9 scores. Such examples include the many traits of lower socioeconomic status, including less education, lack of insurance, unemployment, and being unmarried [30]. Bonfim et al. (2013) demonstrated that Brazilian adults with the worst social indicators, such as low education and income, experienced the most severe periodontal conditions ​​​[31]. These observations align with broader findings linking systemic inequities to adverse health outcomes. The similarities observed between the two studies raise questions about a link between PHQ-9, depression, and periodontitis. Theoretically, periodontitis may also be associated with depression as defined by PHQ-9 due to their commonality of overlapping risk factors determined by SDoH.

On a global scale, age and environment remain pivotal in understanding the influence of SDoH. In South Korea, middle-aged men and women exhibited differing risk profiles: men’s depressive symptoms were more influenced by oral health conditions, while women’s were shaped by chewing difficulties and periodontal disease [32,33]. Although age and sex findings are opposite to those of Califf et al. [30], the content of these two South Korean studies is consistent with the literature findings thus far. Additionally, a German study of older adults identified significant associations between periodontitis, depression severity, and inflammatory biomarkers such as IL-6 and CRP, indicating the physiological underpinnings of these conditions [15].​​ This complexity is further illustrated by research in Chile, which found higher odds of incident depression among edentulous individuals, particularly women, over longitudinal follow-ups, tying oral health directly to mental health outcomes [34].

One study that specifically focused on women was conducted by Naveed and Ramesh, who analyzed periodontal, anxiety, and depression factors for pregnant women [35]. Via the PHQ-9 and Beck Anxiety Inventory (BAI), they found a statistically significant positive correlation with both anxiety and depression scores for periodontal parameters, including plaque index, gingival index, probing pocket depth, and clinical attachment loss. Finally, a recent 2023 study conducted in Mexico found moderate periodontitis to be associated with no schooling and poor oral hygiene, while severe periodontitis was associated with both age and the interaction term of rural area and indigenous origin [36].

The interplay between SDoH, systemic inflammation, oral health, and mental health underscores the need for holistic healthcare models. Addressing inequities in access to dental care and mental health services can mitigate disparities rooted in social and environmental factors. We hypothesize that introducing the Renew Procedure to a qualified patient would improve their oral health by eliminating chronic inflammation and periodontitis. This would effectively decrease the patients’ dissatisfaction levels as represented by the PHQ-9 survey, further augmenting the beneficial qualities of the surgery. While the medical and dental benefits of the Renew Procedure are well-documented [1], this study uniquely focuses on its psychological impact, evaluating how full-mouth rehabilitation affects self-reported mood, well-being, and quality of life as measured by PHQ-9.

## Materials and methods

**Figure 1:**
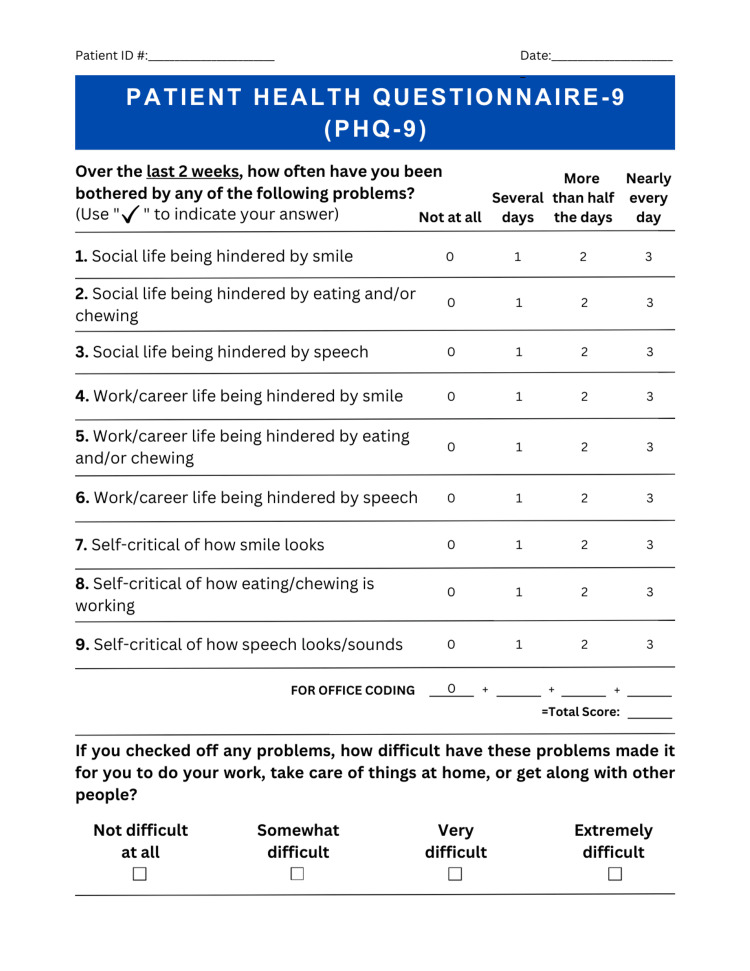
Patient Health Questionnaire-9 (PHQ-9) Created by the authors

The subjects were recruited in the Institutional Review Board (IRB)-approved study (Sterling IRB ID: 11273-WBalanoff) if they received an elective surgery to improve their oral health. Patients were gathered over a span of 12 months (November 2023-November 2024) as more PHQ-9 questionnaires were administered over time. According to the Renew Procedure, all patients had the necessary medical forms and authorizations completed; informed consent and release of records forms allow their de-identified personal medical and health data to be utilized in Health Insurance Portability and Accountability Act (HIPAA)-protected studies to ascertain the health benefits and patient satisfaction of the Renew Procedure. Pre-surgery, during the initial comprehensive examination, patients completed the initial PHQ-9 form, which was modified to include questions regarding SDoH and both dental and personal satisfaction with the surgery (see Figure 1 in Appendices). The same PHQ-9 questionnaire was filled out a second time at the end of the surgical process, typically 4-6 months post-surgery, once healing was completed and patients returned for their final fitting appointment(s).

Patients eligible for inclusion in the PHQ-9 study met specific eligibility criteria. First, these patients were at least 18 years of age at the time of surgery, with no upper age limit. They were scheduled to undergo their surgeries sometime after the introduction of the PHQ-9 questionnaires into the continuum of care at Renew LLC (November 2023). After completing surgery and healing, patients were given a post-surgery PHQ-9 at their final fitting appointment, sometimes referred to as the final delivery appointment. Adult patients eligible for the study had the proper forms and authorizations on file, a pre-surgery PHQ-9, a completed and successful surgery, and a post-surgery/post-healing PHQ-9.

Following the introduction of the PHQ-9 forms and the application of the inclusion criteria, it took 12 months to accumulate N=108 patients. Some patients were missing a post-surgery PHQ-9 in their files and had to be contacted via telephone to complete the questionnaire with medical/dental staff through personal communication. Exclusion criteria included patients who willingly declined participation in the optional PHQ-9 forms during their healthcare visits, resulting in a final sample population of n=103. Medical records were reviewed by research analysts employed at Renew LLC, and data were analyzed by the principal investigator (PI). Paper, digital, and scanned paper medical records were reviewed, but collected data were stored solely electronically in Excel spreadsheets (Microsoft Corp., Redmond, USA) without identifying information, which was stored on a separate password-protected spreadsheet without medical information. Only the PI had access to the identifying personal information.

The patients who met all inclusion criteria were entered into a spreadsheet without personal identification. Initially, the exact date of surgery for each patient was recorded, along with the dates of both the pre- and post-op PHQ-9 forms. The individual answers and aggregate totals for each pre- and post-op PHQ-9 were also documented in the spreadsheet, allowing researchers to view the data as a complete result of all nine questions, while also permitting a more focused analysis on individual questions, if needed.

## Results

In an attempt to fully understand the effects of the Renew Procedure on patients’ health and wellness, a comparative analysis of pre- and post-surgery PHQ-9 scores was conducted. The Renew PHQ-9 was modified to be dental-specific while simultaneously providing insights into a patient’s subjective experience with the Renew Procedure from pre- to post-op. Nearly the entire study sample (103 out of 108 patients) was considered to provide a more in-depth overview of the broad impact of the Renew Procedure on PHQ-9 scores. Subsequently, the data were categorized according to whether the PHQ-9 scores improved, worsened, or stayed the same from pre- to post-op. This was crucial for identifying specific aspects of the Renew Procedure's continuum of care that may have contributed to some patients not having a satisfactory experience. The main goal of the analysis was to uncover the potential relationship between an objectively successful medical procedure that improves overall body health and the subjective experience of the patient undergoing it. The observed pre- and post-op differences were compared to measure this disparity, similarly to how the standard PHQ-9 is assessed over time.

For n=103 patients, 76 had ‘better’ scores (post-op PHQ-9 had a lower total than pre-op PHQ-9). Sixteen had ‘worse’ scores, and 11 showed no change in their scores before and after receiving the Renew Procedure. Table 1 provides an overview of the sample by total population (all) and subgroups (satisfied, unsatisfied, no change). Initial pre-surgery PHQ-9 scores for all patients ranged across the entire possibility of scores (0-27), with 32% exceeding the pre-established threshold of 20 or more. Post-surgery scores also ranged from 0 to 27, but only 9.7% had scores of 20 or above. Furthermore, of these 10 post-surgery scores at 20 or above, two resulted from a net score change of 0, while only six patients in this range obtained worse scores. Average pre- and post-op scores were 13.8 and 5.5, respectively, indicating that the average patient improved their score by 8.3 points (out of 27 total, 30.7%). Table 2 in the Appendices provides an overview of patient scores pre- and post-op.

**Table 1:** Pre- and post-op Patient Health Questionnaire-9 (PHQ-9) averages

	All	Satisfied	Unsatisfied	No change
Pre- and post-op PHQ-9 (N)	103	76	16	11
Average pre-op scores	13.72	15.70	9.75	5.82
Average post-op scores	5.50	3.20	16.19	5.82
Pre- to post-op difference	-8.22	-12.50	6.44	0.00

To gain a better understanding of the 16 patients whose PHQ-9 scores worsened, these patients were contacted by phone. Among those 16 patients, eight explained some version of “getting used to” the new prosthetics in their mouth, taking longer than anticipated; three expressed dissatisfaction with the final outcome of the Renew Procedure; and five could not be reached for comment. Despite providing patients with a new smile and teeth after years (for some) of edentulism or partial edentulism, the Renew prosthetic appliance (Renew LLC, Centennial, USA) has a bite force that is 20% less than the natural bite force of the jaw. This leads some patients to realize that they still face functional deficiencies, highlighting the need for more effective communication with patients during the pre-op stages about what a ‘realistic outcome’ of the Renew Procedure is, compared to the outcome they may be envisioning for themselves. 

## Discussion

The results of this study highlight the intricate relationship between oral health and psychological well-being. By effectively managing chronic periodontal infections, the Renew Procedure appears to significantly reduce systemic inflammation and enhance patient-reported outcomes. Among the 103 patients included, approximately 74% (76 patients) reported lower PHQ-9 scores after the procedure. This outcome aligns with emerging evidence suggesting a connection between severe periodontitis, depression, and systemic inflammation, which indicates a shared inflammatory pathway where chronic oral inflammation may contribute to broader systemic inflammation, potentially exacerbating depressive symptoms [37,38]. By resolving periodontal disease, the Renew Procedure likely interrupts this pathway, reducing overall inflammation and consequently alleviating mood disturbances.

Beyond clinical indicators of success, such as infection control and implant integration, patient satisfaction and quality of life remain essential outcomes. However, not all patients experienced improved mood or satisfaction following treatment: 15.5% (16 patients) reported higher PHQ-9 scores post-procedure, and an additional 10.7% (11 patients) showed no change. This underscores the inherently subjective nature of recovery. Some patients may have faced unrealistic expectations or encountered difficulties adjusting to their new prosthetic teeth. Anecdotal reports revealed frustration among several patients who felt challenged by the initial adaptation period, despite objectively improved oral function. Previous studies confirm that while implant-supported prostheses significantly improve masticatory function and comfort compared to traditional dentures, they may not fully restore a sense of normalcy, particularly in individuals with longstanding tooth loss and jawbone resorption [39,40]. Such constraints, combined with high pre-surgical expectations, can create a disparity between anticipated and actual experiences, leading to emotional dissatisfaction despite clinical success. Managing patient expectations through comprehensive pre-surgical counseling and providing thorough post-operative support, including dietary guidance and prosthetic adjustments, is therefore crucial.

The varied patient outcomes observed may also reflect broader social and economic factors. SDoH, such as income level, educational background, and healthcare access, significantly influence both oral and mental health outcomes [41]. Patients with lower socioeconomic status or less education tend to have higher rates of untreated dental diseases and higher prevalence of depression [42]. These underlying social and economic conditions could affect how patients recover and adapt following procedures like the Renew Procedure. For instance, patients experiencing financial stress or lacking social support may struggle to fully realize the psychological benefits of improved oral health. This suggests clinical interventions alone may be insufficient, emphasizing the importance of integrating broader, more holistic approaches into patient care. Addressing barriers such as transportation, health literacy, and affordability of ongoing care could amplify the positive mental health impacts of oral rehabilitation. Additionally, targeted social support for disadvantaged patients might yield disproportionate benefits, especially if implemented alongside medical treatments.

This study provides important insights into the relationship between oral rehabilitation and mental health, but has notable limitations. For instance, the short follow-up period might have captured only initial mood improvements rather than longer-term adjustments. Some patients may experience delayed benefits as they adapt fully to their prosthetic devices, which the present study may not fully reflect. Additionally, while the PHQ-9 is a validated depression assessment tool, it does not capture other important psychological dimensions such as anxiety, self-esteem, or social functionality. Future research should address these limitations by employing longitudinal controlled trials, ideally over extended periods and with comparison groups receiving alternative or delayed treatments. Incorporating inflammatory biomarkers alongside psychological assessments would also further clarify the mechanistic pathways involved. Qualitative research methods, such as patient interviews, could provide deeper insights into patient experiences and expectations beyond quantitative scores. Additionally, future analyses should stratify outcomes based on SDoH, potentially revealing specific groups that might benefit from supplementary interventions. Overall, a growing body of evidence suggests a bidirectional relationship between oral and mental health [43,44], and continued research should explore integrated care approaches that combine periodontal treatment with mental health support. Ultimately, robust, high-quality studies are essential to confirm causality and inform best practices in comprehensive oral healthcare.

## Conclusions

Periodontal disease has far-reaching effects beyond oral health, contributing to systemic inflammation and mental health issues. Research has established that SDoH, such as socioeconomic status, education, and access to care, intersect with periodontal disease and mental health outcomes. Lower income, limited education, and minority status are associated with worsened periodontal health and higher depression scores. The Renew Procedure has proven to be the first dental procedure that directly affects the overall medical health of patients with type II diabetes, with groundbreaking research demonstrating that removing chronic oral infections, such as periodontal disease, from a patient’s mouth improves the body’s management of hemoglobin A1C. Moreover, the ability to chew a healthier diet allows for a more balanced intake of foods. The social implications of this development are extensive and promising because populations that have traditionally been underserved, including Medicaid recipients and other marginalized groups, now have unprecedented opportunities to enhance various aspects of their lives through improved oral health. For state and federal government agencies, the benefits could also be substantial, as healthier dental conditions correlate directly with reduced emergency room visits for dental pain, lower incidence of related systemic diseases such as diabetes, diminished depression associated with tooth loss, and greater employment opportunities stemming from increased self-esteem. Collectively, these improvements have the potential to positively impact millions around the world. 

## Appendices

**Table 2:** Full Patient Health Questionnaire-9 (PHQ-9) answers

Patient	Pre-surgery answers										Post-surgery answers										Change
	Q1	Q2	Q3	Q4	Q5	Q6	Q7	Q8	Q9	Total	Q1	Q2	Q3	Q4	Q5	Q6	Q7	Q8	Q9	Total	
1	3	3	2	3	3	3	3	3	3	26	0	0	0	0	0	0	0	0	0	0	-26
2	2	0	2	2	0	1	3	3	3	16	0	0	0	0	0	0	0	0	0	0	-16
3	3	1	3	1	1	1	3	3	3	19	0	0	0	0	0	0	0	0	0	0	-19
4	3	3	3	3	3	3	3	3	3	27	0	0	0	0	0	0	0	0	0	0	-27
5	0	1	3	3	3	3	3	3	3	22	0	0	0	0	0	0	0	0	0	0	-22
6	3	1	0	3	0	2	3	0	0	12	0	0	0	0	0	0	0	0	0	0	-12
7	3	2	3	3	2	3	3	3	3	25	0	1	0	0	0	0	3	3	3	10	-15
8	0	1	0	0	1	0	3	3	3	11	0	1	0	0	1	0	0	1	1	4	-7
9	0	3	1	0	1	0	2	2	2	11	0	0	0	0	0	0	0	0	0	0	-11
10	0	0	0	0	0	0	3	1	1	5	0	0	0	0	0	0	0	0	0	0	-5
11	3	3	3	2	2	2	3	3	3	24	3	2	3	3	2	3	3	2	3	24	0
12	0	0	0	0	3	0	1	2	1	7	0	0	0	0	0	0	0	0	0	0	-7
13	0	3	0	0	3	0	2	3	0	11	0	1	0	0	1	0	0	1	0	3	-8
14	0	3	0	0	3	0	2	3	0	11	1	1	1	1	1	1	1	1	1	9	-2
15	3	3	3	3	3	3	3	3	3	27	3	3	3	0	3	2	0	3	3	20	-7
16	0	0	0	0	0	0	1	1	0	2	0	0	0	0	0	0	1	0	0	1	-1
17	3	2	2	3	3	2	3	3	2	23	0	1	0	0	1	0	0	1	0	3	-20
18	1	3	0	0	3	0	3	3	0	13	0	0	0	0	0	0	0	0	0	0	-13
19	3	3	2	3	3	2	3	3	3	25	0	1	0	0	0	0	0	1	1	3	-22
20	0	3	0	0	3	0	2	2	0	10	0	1	0	0	1	0	0	1	0	3	-7
21	0	0	0	0	0	0	0	3	0	3	0	0	0	0	0	0	0	0	0	0	-3
22	0	0	0	0	0	0	3	2	0	5	2	3	2	0	3	2	0	2	1	15	10
23	3	0	0	0	0	0	2	1	0	6	2	2	1	1	2	1	2	2	2	15	9
24	1	3	1	1	2	0	2	3	1	14	0	1	1	0	0	0	1	2	2	7	-7
25	2	3	0	0	0	0	3	3	0	11	1	3	1	0	0	0	3	2	1	11	0
26	3	3	0	2	1	0	3	2	0	14	1	2	1	0	0	1	1	1	1	8	-6
27	0	0	0	0	0	0	0	0	0	0	0	0	0	0	0	0	0	0	0	0	0
28	0	1	2	0	0	0	0	0	2	5	0	0	0	0	0	0	0	0	0	0	-5
29	3	3	3	2	3	3	3	3	3	26	1	1	1	0	1	1	2	2	2	11	-15
30	0	0	0	1	0	0	0	0	0	1	0	0	0	0	0	0	0	0	0	0	-1
31	0	3	3	3	3	3	3	3	2	23	0	0	0	0	0	0	0	0	0	0	-23
32	0	1	1	0	0	0	0	1	1	4	0	1	1	0	0	1	0	1	1	5	1
33	3	3	3	3	3	3	3	3	3	27	3	3	2	3	3	2	3	3	3	25	-2
34	3	2	0	3	3	3	3	3	0	20	0	0	0	0	0	0	0	0	0	0	-20
35	3	3	2	3	3	2	3	3	2	24	1	1	1	1	1	1	1	1	1	9	-15
36	3	3	2	2	2	2	2	3	2	21	3	3	3	3	3	3	3	3	3	27	6
37	3	3	0	3	3	3	3	3	0	21	0	1	0	0	0	0	0	0	0	1	-20
38	3	3	0	1	1	0	3	2	0	13	3	3	0	2	2	2	3	1	2	18	5
39	0	3	0	0	0	3	0	0	0	6	0	1	0	0	1	0	0	0	0	2	-4
40	0	0	0	0	0	0	0	0	0	0	0	0	0	0	0	0	0	0	0	0	0
41	1	1	1	1	1	1	1	1	1	9	1	3	3	3	3	2	2	3	3	23	14
42	0	0	0	0	0	0	3	2	0	5	0	0	0	0	0	0	0	0	0	0	-5
43	1	3	0	0	0	0	3	3	3	13	0	0	0	0	0	0	0	0	0	0	-13
44	0	1	0	0	1	0	3	1	0	6	0	0	0	0	0	0	0	0	0	0	-6
45	1	0	0	0	0	0	2	1	0	4	0	0	0	0	0	0	0	0	0	0	-4
46	0	0	0	0	0	0	3	0	0	3	0	1	1	0	1	1	0	1	1	6	3
47	0	0	0	0	0	0	3	2	0	5	0	0	0	0	0	0	0	0	0	0	-5
48	3	3	2	3	3	2	3	3	3	25	0	0	0	0	0	0	0	0	0	0	-25
49	3	3	0	3	3	3	3	3	0	21	0	1	0	0	0	0	0	3	0	4	-17
50	3	3	3	0	0	0	3	3	0	15	3	3	3	3	2	2	3	2	2	23	8
51	3	3	3	3	3	3	3	3	3	27	0	0	0	0	0	0	0	0	0	0	-27
52	0	0	0	0	0	0	0	0	0	0	0	0	0	0	0	0	0	0	0	0	0
53	3	3	3	0	0	0	0	3	3	15	0	3	2	0	3	0	0	3	2	13	-2
54	3	3	3	3	3	3	3	3	3	27	0	0	0	0	0	0	0	0	0	0	-27
55	3	3	3	3	3	3	3	3	3	27	0	1	0	0	1	0	0	1	0	3	-24
56	3	2	0	0	0	0	3	3	2	13	0	0	2	0	0	0	0	0	2	4	-9
57	0	2	0	0	2	0	0	2	0	6	0	0	0	0	0	0	0	0	0	0	-6
58	0	0	0	0	0	0	2	3	2	7	0	0	0	0	0	0	0	0	0	0	-7
59	0	1	0	0	1	0	1	1	0	4	0	2	0	0	0	0	0	2	0	4	0
60	3	2	0	1	1	0	3	3	2	15	1	1	0	0	0	0	0	1	0	3	-12
61	0	0	0	0	0	0	0	0	0	0	0	0	0	0	0	0	0	0	0	0	0
62	0	0	0	0	0	0	0	0	0	0	0	1	0	0	1	0	0	1	0	3	3
63	0	1	0	0	2	1	0	0	1	5	1	0	0	0	1	1	0	0	0	3	-2
64	1	3	0	1	1	0	2	3	1	12	0	3	0	0	1	0	0	3	0	7	-5
65	1	1	0	0	3	0	3	2	0	10	0	0	0	0	0	0	0	0	0	0	-10
66	3	3	3	2	2	2	3	3	3	24	3	3	3	3	3	3	3	3	3	27	3
67	0	0	0	0	0	0	0	0	0	0	0	0	0	0	0	0	0	0	0	0	0
68	3	0	0	3	0	0	3	0	0	9	3	3	3	0	0	0	3	3	3	18	9
69	3	3	3	0	0	0	3	3	3	18	0	0	0	0	0	0	0	0	0	0	-18
70	0	0	0	2	0	0	2	0	0	4	0	0	0	0	0	0	0	0	0	0	-4
71	1	2	1	1	2	2	2	1	0	12	1	3	0	1	3	0	3	3	3	17	5
72	0	0	0	0	0	0	0	0	0	0	0	0	0	0	0	0	0	0	0	0	0
73	3	3	3	3	3	3	3	3	3	27	2	2	2	2	2	2	2	2	2	18	-9
74	3	3	3	3	3	3	3	3	3	27	0	0	0	0	0	0	0	0	0	0	-27
75	0	0	0	0	0	0	0	0	0	0	0	0	0	0	0	0	0	0	0	0	0
76	2	0	0	3	0	0	3	1	0	9	0	0	0	0	0	0	0	0	0	0	-9
77	3	3	0	2	3	0	3	3	3	20	0	0	0	0	0	0	0	0	0	0	-20
78	0	0	0	0	0	0	3	0	0	3	0	3	0	0	0	0	0	3	0	6	3
79	3	3	0	0	2	0	3	3	0	14	3	3	3	3	3	3	3	3	3	27	13
80	3	3	3	0	0	0	1	1	1	12	0	0	0	0	0	0	0	0	0	0	-12
81	3	3	3	3	3	3	3	3	3	27	0	1	0	0	1	1	0	1	1	5	-22
82	3	3	3	3	3	3	3	3	3	27	0	1	1	0	1	1	0	1	1	6	-21
83	2	3	3	2	3	3	0	3	3	22	0	1	3	1	3	1	3	3	3	18	-4
84	2	2	1	1	1	1	1	1	1	11	0	0	0	0	0	0	0	0	0	0	-11
85	0	0	0	0	0	0	3	3	3	9	0	0	0	0	0	0	0	0	0	0	-9
86	0	0	0	0	0	0	0	0	0	0	1	2	0	1	1	0	0	1	1	7	7
87	1	1	1	1	1	1	1	1	1	9	0	0	0	0	0	0	0	0	0	0	-9
88	0	1	1	0	0	0	3	3	1	9	0	0	0	0	0	0	0	0	1	1	-8
89	3	3	2	2	3	2	3	3	1	22	0	0	0	0	0	0	0	0	0	0	-22
90	2	3	0	2	3	0	3	3	0	16	0	1	0	0	1	0	1	0	0	3	-13
91	2	3	2	0	0	1	1	2	1	12	0	1	1	0	1	1	1	2	1	8	-4
92	3	3	2	2	3	2	2	3	2	22	0	0	0	0	0	0	0	0	0	0	-22
93	3	3	2	3	3	2	3	3	3	25	0	0	0	0	0	0	0	0	0	0	-25
94	3	2	2	1	1	1	3	3	2	18	0	0	0	0	0	0	0	0	0	0	-18
95	3	3	2	1	3	1	3	3	3	22	3	3	3	0	0	0	3	3	3	18	-4
96	3	3	2	3	3	2	3	3	3	25	2	3	3	2	3	3	3	3	3	25	0
97	1	1	1	1	1	1	1	1	1	9	0	0	0	0	0	0	0	0	0	0	-9
98	3	3	1	0	0	0	3	3	2	15	0	0	0	0	0	0	0	0	0	0	-15
99	1	3	2	2	3	2	1	3	2	19	0	1	0	0	1	0	0	1	1	4	-15
100	3	3	3	3	3	3	3	3	3	27	0	0	0	0	0	0	0	0	0	0	-27
101	0	2	0	0	0	0	3	2	0	7	0	0	0	0	0	0	0	0	0	0	-7
102	3	3	0	3	3	0	3	3	0	18	1	1	0	1	1	0	1	1	0	6	-12
103	3	3	0	3	3	0	3	3	0	18	3	2	2	3	3	2	3	2	2	22	4
